# Susceptibility of Flordaguard peach rootstock to a resistant-breaking population of *Meloidogyne floridensis* and two populations of *Meloidogyne arenaria*

**DOI:** 10.21307/jofnem-2021-111

**Published:** 2022-01-21

**Authors:** Sai Qiu, Mary Ann D. Maquilan, Jose X. Chaparro, Janete A. Brito, Thomas G. Beckman, Donald W. Dickson

**Affiliations:** 1 Entomology and Nematology Department, University of Florida, Gainesville, FL 32611; 2 Horticulture Science Department, University of Florida, Gainesville, FL 32611; 3 Nematode Diagnostic Laboratory, Florida Department of Agriculture and Consumer Services, Division of Plant Industry, Gainesville, FL 32608; 4 USDA ARS, Southeastern Fruit and Tree Nut Research Laboratory, Byron, GA

**Keywords:** Flordaguard, *Meloidogyne arenaria*, *Meloidogyne floridensis*, Peach rootstock, *Prunus persica*, Root-knot nematode

## Abstract

Cultivar Flordaguard is suggested as a root-knot nematode (RKN) resistant rootstock for Florida peaches, however, RKN disease has been observed on this rootstock in peach orchards. Our goal was to confirm whether the RKN resistance breaking isolates of *M*. *floridensis* and *M*. *arenaria* indeed could infect and reproduce on the peach rootstock cv. Flordaguard in both laboratory and field studies. Root galling occurred on all peach cultivars evaluated including Flordaguard, Flordaglo, Okinawa, and Lovell, in the presence of the RKN resistance-breaking isolates of *M. floridensis* (MfGnv14) and two *M. arenaria* isolates (Ma1 and Ma2). These rootstocks showed varying degrees of susceptibility (to a lesser extent in Okinawa) to these three RKN resistance-breaking isolates. The importance of nematode inoculum concentrations in differentiating between resistance and susceptible plants was demonstrated, and thus are an important factor to consider in nematode resistance breeding programs. In host differential tests the peach-originated isolates of *M. floridensis* and *M. arenaria* behaved similarly with the vegetable-originated isolates of *M. floridensis* on tomato, peanut, watermelon, and tobacco, but showed variable host responses on cotton and pepper. The two *M. arenaria* isolates from peach reproduced on pepper but not on peanut. To our knowledge this is the first report of *M*. *arenaria* race 3 infecting Flordaguard and pepper in Florida. Soil and root samples collected from cv. Flordaguard infected trees at two commercial peach orchards showed that *M*. *floridensis* and *M*. *arenaria* were established on the rootstock.

Florida growers are considering peach (*Prunus persica* [L.] Batsch) (Sharpe, 1967) as a viable alternative crop because of the availability of several quality low-chill peach cultivars well-adapted to the subtropical climate and the unique advantage of an early-season market (Olmstead et al., 2015). Peach rootstocks with root-knot nematode (RKN) resistance, a necessary component for the productivity and longevity of an orchard, have primarily been developed in rootstock breeding programs. The peach rootstock cvs. Nemaguard (*Prunus persica* x *P. davidiana*) and Nemared (*P. persica* x *P. davidiana*), developed by the U.S. Department of Agriculture, and Okinawa, introduced as seed from Japan, were reported resistant to *M. incognita, M. javanica*, and *M. arenaria* (Sharpe, 1957; Okie et al., 1985; Esmenjaud et al., 1997). Okinawa and Nemaguard were planted extensively in Florida during the 1960’s because they were considered to have a high degree of resistance to the prevalent RKN species *M. incognita* and *M. javanica* (Sherman and Lyrene, 1983). These rootstocks, including Nemared, were eventually found to be susceptible to a RKN population in North Florida. Initially the nematode was misidentified as *M. incognita* race 3 (Sharpe, 1967; Sherman and Lyrene, 1983), however, further investigations led to it being identified as a new species, *M. floridensis*, common name, the peach RKN (Handoo et al., 2004).

Since its introduction in 1991, the peach seedling rootstock cv. Flordaguard was reported as resistance to all *Meloidogyne* spp. considered a threat to the peach industry in Florida and was suggested to become the standard rootstock for commercial low-chill peach production replacing Okinawa and Nemaguard (Sherman et al., 1991). After Flordaguard was released as the suggested RKN resistant rootstock, there were reports of it being damaged by *Meloidogyne* spp. (Brito et al., 2015, 2016). Although limited information is available, recent field surveys carried out in commercial orchards showed that the rootstock was infected by *M. floridensis* and *M. arenaria* (Brito et al., 2016). Because Flordaguard is a seedling-propagated rootstock there was speculation that outcrossing resulted in loss of resistance (Nyczepir et al., 2006). Certainly, that could have happened in some orchards but in two commercial orchards where trees were infected by *M*. *floridensis* and *M*. *arenaria* numerous trees had the characteristic red-leaf stems growing off the rootstock, which suggested the rootstock was true to variety. Furthermore, an isolate of *M. floridensis*, MFGnv14, was reported inducing severe root galling and reproducing on Flordaguard (Maquilan et al., 2018a). Most recently *M*. *floridensis* also was reported causing decline on RKN resistant peach-almond hybrids, Hansen 536 and Bridgt’s Hybrid^®^5 rootstocks in California almond orchards (Westphal et al., 2019), and peach rootstock cv. Guardian in South Carolina (Reighard et al., 2019).

*Meloidogyne floridensis* is being considered an emerging pathogen because of its ability to reproduce on RKN resistant plant species, including peach (*RMia* and *RMja* genes), peach-almond hybrid rootstocks, pepper (*N* gene), tomato (*Mi-1* gene), and tobacco cv. NC 95 (*Rk1* gene) (Stanley et al., 2009; Maquilan et al., 2018a; Marquez et al., 2021).

*Meloidogyne arenaria* was also found infecting Flordaguard peach trees in established orchards in Florida. The nematode was causing severe root galling, plant dieback, and stunted growth (Brito et al., 2016; Dickson and Chaparro, pers. comm.). Polyacrylamide gel electrophoresis analyses revealed these *M. arenaria* populations having an esterase and malate dehydrogenase phenotype identified as EST = A2 and MDH = N3, respectively. These phenotypes differ from the more commonly encountered *M*. *arenaria* race 1 phenotype EST = A2; MDH = N1. Phylogenetic analysis performed on the peach infecting *M*. *arenaria* populations assigned them as H3 phenotype (Brito et al., 2016).

Our objectives were to: (i) determine infectivity and reproduction on RKN resistant and susceptible peach rootstock cultivars at different inoculum levels of a resistance-breaking *M. floridensis* isolate; (ii) determine the infectivity of *M. floridensis* and *M. arenaria* isolates to three peach rootstock cultivars; (iii) confirm the susceptibility of the resistant Flordaguard rootstock to the resistance-breaking nematode isolate, *M. floridensis*, and the two *M. arenaria* isolates; iv) conduct differential host tests of four *M. floridensis* and three *M. arenaria* isolates; and v) confirm infection of Flordaguard rootstock by *M. floridensis* and *M*. *arenaria* populations in two commercial peach orchards.

## Materials and methods

### Nematode isolates

Isolates of *M. floridensis* and *M. arenaria* used in this study originated from various host crops in several Florida counties (Table 1). Three isolates, Mf1, Mf2, and Mf4 were acquired from the nematode collection of the Division of Plant Industry, Florida Department of Agriculture and Consumer Services, Gainesville, and were characterized using both biochemical and DNA analyses (Brito et al., 2008; Smith et al., 2015). The *M. floridensis* isolate, Mf4, originated from peach Nemaguard, University of Florida (UF) stone fruit breeding orchard, Gainesville, FL, and is the topotype described by Handoo et al. (2004). Isolate Mf6 originated from a peach orchard located near Ft. Pierce, St. Lucia Co., FL. Another *M*. *floridensis* isolate (MfGnv14) originated from a severely galled cv. Flordaguard rootstock found growing in the UF stone fruit orchard. This isolate was used subsequently for genetic studies of resistance in peach rootstocks (Maquilan et al., 2018a, 2018b), as well as comparative genomics of RKN (Szitenberg et al., 2017). The MfGnv14 inoculum used for the current study was collected after two cycles (6 months) of propagation on tomato, whereas the other *M*. *floridensis* isolates were maintained over 10 years on greenhouse grown tomato. *M. arenaria* Ma1 and Ma2 were isolated from commercial peach orchards in Florida in 2014. The *M. arenaria* (Ma3 isolate) originated from Flordaguard rootstock grown at a commercial orchard located near Auburndale, Polk Co., FL. Each isolate was reared from a single egg mass and cultured on tomato (*Solanum lycopersicum* L. cv. Agriset 334) at 21°C ± 10°C in the greenhouse.

**Table 1.** Origins of *Meloidogyne floridensis* and *M. arenaria* isolates collected from different agricultural sites in Florida.

Nematode species	Nematode isolates (Accession no.)^a^	Original host	Location	References
*M. floridensis*	Mf1 (N05-227-17B)	Tomato	Seminole Co	Brito et al. (2008), Smith et al. (2015)
	Mf2 (N03-1582-2B)	Tomato	Indian River, Co.	Brito et al. (2008), Stanley et al. (2009)
	Mf4 (N03-1894)	Nemaguard peach	Alachua Co.	Handoo et al. (2004), Brito et al. (2008), Stanley et al. (2009)
	Mf6	Flordaguard peach	St. Lucie Co.	Present study^b^
	MfGnv14	Flordaguard peach	Alachua Co.	Maquilan et al. (2018a, 2018b) ^c^
*M. arenaria*	Ma1 (PS-1567)	Flordaguard peach	Polk Co.	Unpubl. data^c^
	Ma2 (PS-19)	Flordaguard peach	Polk Co.	Unpubl. data^c^
	Ma3	Flordaguard peach	Polk Co.	Present study^b^

Notes: ^a^RKN collection number, Division of Plant Industry, Florida Department of Agriculture and Consumer, Gainesville, FL. Services-DPI, FDACS. ^b^Nematodes were extracted from root samples collected while monitoring nematode population densities in two commercial peach orchard sites. ^c^Collected in 2014 during a nematode survey of peach orchards in Florida, a project supported by Florida Department of Agriculture and Consumer Services, Specialty Crop Block Grant No. 20727.

For all experiments nematode eggs and juveniles were extracted from infected tomato roots using 0.25% NaOCl (Hussey and Barker, 1973), as modified (Boneti and Ferraz, 1981). The nematode suspensions were used immediately to inoculate peach seedlings. In all peach experiments, tomato cv. Agriset 334 was included as a control to monitor inoculum viability. All plants in experiments were watered daily and fertilized weekly (4.5 g/liter of 24N-8P-16K Miracle Gro; Scotts Miracle-Gro Products) (Marysville, OH).

### Resistant-breaking *M. floridensis* isolate on peach rootstocks at three inoculum levels

The infectivity and reproduction of the resistance breaking *M*. *floridensis* isolate were determined. The MfGnv14 isolate was applied at 2,000, 5,000, and 10,000 eggs on Flordaguard and Okinawa (both RKN resistant), and a RKN susceptible peach scion cv. Flordaglo. Flordaglo was included to ensure the infectivity of the nematode inoculum on peach. Cuttings, ca. 15 cm long, of each of the three peach cultivars were prepared for rooting. The cuttings were placed in growing trays containing steam-pasteurized 3:1 mixture of peat-based soil mix (Fafard 2B; Sun Gro Horticulture) (Agawam, MA) and perlite, and allowed to root under a mist propagation system in the greenhouse. After 3 months under the mist system, the rooted cuttings were transplanted into 16-cm-diam. clay pots filled with steam-pasteurized peat and kept in the greenhouse with regulated air temperatures (21 ± 8°C) over the winter. After 6 months (during spring), rooted cuttings were transplanted into 25-cm-diam. clay pots filled with 3:1 mixture of steam-pasteurized sand and perlite. After 1 month, the nematode inoculum was applied into four 3-cm-deep punched holes around the stem base and then pinched closed. The two-factor (inoculum densities and cultivars) factorial designed experiment with four replicates was placed on greenhouse benches in a randomized complete block design. The experiment was terminated 60 days after nematode inoculation.

### Infectivity of *M. floridensis* and *M. arenaria* isolates on three peach rootstocks

Galling and reproduction were determined of three *M. floridensis* isolates Mf1, Mf2, Mf4, and two *M. arenaria* isolates Ma1, Ma2 on two RKN resistant peach rootstocks Flordaguard, Okinawa, and the RKN susceptible cv. Lovell. Lovell was included to verify the infectivity of the nematode isolates on peach. All seedlings were produced from seed (Blaker et al., 2013, Souza et al., 2017). After 60 to 90 days, germinated seeds were sown in growing trays filled with steam-pasteurized peat-based soil mix as mentioned above. Seedlings (ca. 20-cm tall) were transplanted into clay pots containing 2:1:1 steam-pasteurized mixture of sand, coarse vermiculite, and perlite. One month later, 4-month-old seedlings were inoculated with 10,000 eggs/plant and maintained for 5 months. The two-factor (isolate and cultivar) experiment was set up on greenhouse benches in a randomized complete block design with seven replicates.

### Confirmation of Flordaguard rootstock susceptibility to the RKN resistance breaking isolates

The susceptibility of Flordaguard (RKN resistant rootstock) to *M. floridensis* isolate MfGnv14 and two *M. arenaria* isolates Ma1 and Ma2 was tested using 20,000 eggs of each nematode isolate per plant. One-year-old Flordaguard rooted cuttings were provided by Island Grove Ag Products, Hawthorne, FL. The seedlings were transplanted into pots filled with 1:4 mixture of sand and peat. The trees from which cutting were obtained were from own-rooted Flordaguard trees grown at the UF Plant Science Research and Education Unit, Citra, FL and had been verified as true to variety based on SSR-marker-based fingerprinting (Maquilan et al., 2018b). The experiment consisted of only a single factor (isolate) with eight replicate plants for each of the three isolates.

### Differential host tests

The race designation of RKN species and isolates (Table 1) was determined by host differentials. The differentials included: tobacco *(Nicotiana tabacum* cv. NC95), cotton (*Gossypium hirsutum* cv. Deltapine 16), pepper (*Capsicum annuum* cv. California Wonder), watermelon (*Citrullus lanatus* cv. Charleston Grey), peanut (*Arachis hypogaea* cv. FL 07), and tomato (cv. Agriset 334). The previously described procedure (Taylor and Sasser, 1978) was modified as follows: freshly hatched and active J2 instead of eggs were used as inoculum to ensure optimal viability. Use of J2 inoculum as an optimum infective stage may resolve conflicting results from previous host-range tests with *M. floridensis* (Handoo et al., 2004; Kokalis-Burelle and Nyczepir, 2004; Stanley et al., 2009) that might be attributed to the variation in the infection rate (or ability) of J2 when using eggs as starting inoculum. The J2 inoculum was applied to three punched holes (3-cm deep) around the stem base to achieve a concentration of 3,000 J2/plant. Each isolate was inoculated onto the six differential hosts and replicated three to five times in a completely randomized design on greenhouse benches. The test was repeated.

Sixty days after inoculation, root systems were removed from the pots, washed, and rated for root galling and egg masses on a 0 to 5 scale (Taylor and Sasser, 1978). Before rating, root systems were soaked in 20% (v/v) red food coloring solution (Chef’s Quality; RD/Jet) (College Point, NY) for 20 min to stain egg masses (Thies et al., 2002). Additionally, because no clear root symptoms and (or) nematode sign were observed on the root systems of both cotton and pepper, eggs were then extracted with 1% NaOCl and the final number of eggs (Pf) per gram of fresh roots was counted for each plant and the reproductive factor (RF = Pf/Pi) determined. Host suitability was designated as follows: RF ≥ 1 = good host; 0.1 < RF < 1.0 = poor host; RF ≤ 0.1 = nonhost (Sasser et al., 1984).

### RKN infection of peach in commercial peach orchards

Two commercial peach orchards discovered to have Flordaguard rootstock infected by RKNs were designated for soil and root sampling, totaling seven sampling collections between April 2015 to July 2016. Both were stated to have been transplanted with peach scions grafted on Flordaguard rootstock. Orchard A was in central Florida near Auburndale, Polk Co.; orchard B was in south Florida near Fort Pierce, St. Lucie Co. Soil at orchard A, classified as Candler sand with a depth well below 1.5 m, whereas soil at orchard B, classified as an Ankona sand with a depth of ca. 0.05 m, and was stratified over a perched water table. Trees at orchards A and B were ca. 5- and 15-year old, respectively. Soil analyses was conducted by the UF Analytical Services Laboratory, Gainesville, FL (data not shown). Orchard A soil was slightly acidic with a pH 6.0 to 6.4, whereas orchard B soil was strongly acidic with a pH 4.3 to 5.5. The total percentage of silt and clay were less than 3% in the soil samples.

At each site and sampling date, four root-knot-nematode-infected trees were arbitrarily selected for collection of root and soil samples based on above ground symptoms that included small tree size, leaf drop, sparse foliage, and crown dieback. Before collecting samples from the top 15 cm of soil and weeds were removed from each sampling site. Samples were collected from the top 50 cm deep layer of soil at three locations around each tree. One liter of soil mixed with peach roots was collected from base of each tree, mixed well and placed in a plastic bag before storing at 10°C until processed (Barker and Nusbaum, 1968). RKN females from galled peach roots were hand-picked and subjected to PAGE for species identification based on esterase (EST) and malate dehydrogenase (MDH) enzymatic profiles (Dickson et al., 1971; Esbenshade and Triantaphyllou, 1985), and DNA analysis when needed (Brito et al., 2016). Nematodes were extracted from 200 cm^3^ of soil by centrifugal-flotation method (Jenkins, 1964). Root samples were weighed, and nematodes extracted as stated above. The numbers of nematodes extracted from the soil as well as J2 and eggs from roots were counted. Also, other plant-parasitic nematodes extracted from soil were identified based on their morphology.

### Data analyses

Data were subjected to two-way analysis of variance (ANOVA) from peach experiments 1 (concentration × cultivar) and 2 (isolate × cultivar), and one-way ANOVA for peach experiment 3 (isolate) using R version 3.3.0 (R-Core Team, 2014) with RStudio version 0.99.903 (RStudio, 2014). Variables subjected to analyses were gall index (GI), egg mass index (EMI), reproductive factor (RF), and eggs per gram fresh roots (EGFR). Data were transformed before statistical analyses as follows: GI and EMI by log_10_ (*x* + 1), RF by fourth root, and EGR by arcsine. Means were separated using Tukey’s honest significant difference (*P* ≤ 0.05). Unless otherwise noted, all differences were considered statistically significant at the 0.05 probability level.

## Results

### Resistance-breaking *M. floridenis* isolate on peach rootstocks using three inoculum levels

*Meloidogyne floridensis* isolate MfGnv14 induced galls and reproduced on RKN resistant rootstocks Flordaguard, Okinawa, and the susceptible Flordaglo. The gall and egg mass indices were less on Okinawa than on Flordaguard and Flordaglo (Table 2). There were less galling and egg masses induced from the low inoculum of 2,000 eggs/plant, whereas there were no differences between the higher inoculum densities of 5,000 and 10,000 eggs. The level of inoculum and rootstock cultivar both affected the degree of infection and reproduction.

**Table 2.** Effect of inoculum concentration and peach cultivars on galling and egg mass of *Meloidogyne floridensis* (MfGnv14) at 60 days after inoculation.

Treatment	Galling index^a^	Egg mass index^a^
Concentration (eggs and J2/plant)		
2,000	2.4b	2.2b
5,000	4.3a	3.6a
10,000	4.2a	3.2a
Cultivar^b^		
Flordaguard	4.3a	3.7a
Okinawa	2.4b	1.3b
Flordaglo	4.2a	4.0a
Tomato cv. Agriset 334	5.00	5.00
Analysis of variance		
Concentration	0.0001***	0.0387*
Cultivar	<0.0001***	<0.0001***
Concentration x cultivar	0.9795 *ns*	0.4377 *ns*

Notes: ^a^Galling (GI) and egg mass indices (EMI): 0 = no galls or egg masses, 1 = 1-2, 2 = 3-10, 3 = 11-30, 4 = 31-100, 5 = ≥ 100 galls or egg masses per plant (Taylor and Sasser, 1978). GI and EMI data were subjected to log_10_ (x + 1) transformation before analysis of variance. Means are average of duplicate tests. Data are non-transformed means of four replicates. Means within a main effect in the same column followed by the same letter are not different (*P* < 0.05) based on Tukey’s honest-significant difference test. ^b^Plant materials were 10-month-old rooted stem cuttings. Tomato cv. Agriset 334 was included to check nematode viability. *ns* = No statistically significant differences among means.

### Infectivity of *M. floridensis* and *M. arenaria* isolates on three peach rootstocks

Among the three peach rootstocks, only the RKN susceptible Lovell was infected by all three *M. floridensis* isolates (Mf1, Mf2, Mf4) (Table 3). These three isolates failed to induce galls and did not reproduce on the RKN resistant Flordaguard, in contrast to the heavy infection induced by the RKN resistance breaking isolate MfGnv14 (Table 2). The three *M*. *floridensis* isolates also did not produce galls and failed to reproduce on Okinawa (Table 3), whereas the *M. arenaria* isolates Ma1 and Ma2 induced galls and egg masses on Flordaguard. On Flordaguard Ma2 induced a higher number of galls and egg masses to a greater extent than Ma1, but this did not correspond to greater egg numbers (Table 3). Only Ma1 was tested on Okinawa, and Lovell because of insufficient inoculum at the time when the plant materials were ready. This isolate produced galls on Okinawa but failed to produce egg masses (Table 3). All isolates infected and reproduced similarly on Lovell.

**Table 3.** Comparison of the degree of galling and reproduction rate of *Meloidogyne floridensis* and *M. arenaria* isolates on peach cvs. Flordaguard, Okinawa, and Lovell after 5 month’s growth under greenhouse conditions.

Cultivar^a^	Isolate	GI^b^	EMI^b^	EGFR^c^	RF^c^
Flordaguard	Mf1	0	0.0	0	0.0
	Mf2	0	0.0	0	0.0
	Mf4	0	0.0	0	0.0
	Ma1	2.9	3.5	377	3.6
	Ma2	5.0	5.0	259	3.4
Okinawa	Mf1	0.0	0.0	0	0.0
	Mf2	0.0	0.0	0	0.0
	Mf4	0.0	0.0	0	0.0
	Ma1	2.6	0.0	0	0.0
Lovell	Mf1	5.0	5.0	1,244	11.8
	Mf2	5.0	5.0	1,604	18.0
	Mf4	5.0	5.0	1,691	14.5
	Ma1	5.0	5.0	1,485	18.0
Tomato	Mf1	5.0	5.0	5,932	42.3
cv. Agriset 334	Mf2	5.0	5.0	7,548	62.7
	Mf4	5.0	5.0	8,915	59.1
	Ma1	5.0	5.0	8,742	42.4

Notes: ^a^Four month-old peach seedlings were inoculated with 10,000 eggs/plant. Peach rootstock cv. Lovell used as a susceptible control and tomato cv. Agriset 334 included to check nematode viability. ^b^Galling (GI) and egg mass indices (EMI) were based on a 0 to 5 scale where 0 = no galls or egg masses, 1 = 1-2, 2 = 3-10, 3 = 11-30, 4 = 31-100, 5 = ≥ 100 galls or egg masses per plant (Taylor and Sasser, 1978). Data were subjected to log_10_ (x + 1) transformation before analysis of variance. Data are non-transformed means of seven replicates. ^c^EGFR = Eggs per gram of fresh root. Reproduction factor (RF) = ratio of nematode eggs at 5 months after inoculation to initial inoculum concentration of 10,000 eggs (Sasser et al., 1984). EGFR and RF data were subjected to arcsine and fourth-root transformation, respectively, before analysis of variance. Data are non-transformed means of seven replicates.

### Confirmation of Flordaguard rootstock susceptibility to the RKN resistance breaking isolates

*Meloidogyne floridensis* isolate MfGnv14, and two *M*. *arenaria* isolates Ma1, Ma2 infected and reproduced on Flordaguard rootstock. There were no differences in galling, egg mass indices, or RF among these three isolates (Table 4).

**Table 4.** Galling and reproduction rates of *Meloidogyne floridensis* isolate MfGnv14 and *M. arenaria* isolates Ma1 and Ma2 applied at 20,000 eggs/plant on 1-year old true-to-variety peach cv. Flordaguard after 5 month’s growth under greenhouse conditions.

Nematode isolate	GI^a^	EMI^a^	EGFR^b^	RF^b^
MfGnv14	3.9	3.8	58	0.46
Ma1	3.1	3.1	65	0.40
Ma2	3.0	3.1	60	0.39
*P-value*	0.375 *ns*	0.547 *ns*	0.5414 *ns*	0.765 *ns*

Notes: ^a^Galling (GI) and egg mass (EMI) indices were based on a 0 to 5 scale where 0 = no galls or egg masses, 1 = 1-2, 2 = 3-10, 3 = 11-30, 4 = 31-100, 5 = >100 galls or egg masses per plant (Taylor and Sasser, 1978). Data were subjected to log_10_ (x + 1) transformation before analysis of variance. Data are non-transformed means of eight replicates. ^b^EGFR = Eggs per gram of fresh root weight. Reproduction factor (RF) = ratio of nematode eggs at 5 months after inoculation to initial inoculum concentration of 20,000 eggs (Sasser et al., 1984). EGFR and RF data were subjected to arcsine and fourth-root transformation, respectively, before analysis of variance. Data are non-transformed means of eight replicates. Means in the same column followed by the same letter are not different (*P* < 0.05) based on Tukey’s honest-significant difference test. *ns* = No statistically significant differences among means.

### Differential host tests

All *M. floridensis* and *M*. *arenaria* isolates infected, induced root galling, and reproduced on tomato, watermelon, and tobacco (GI, EMI = 5), but not on peanut (Table 5). The galls they caused, if any, on cotton and pepper roots were only detectable with aid of a magnifying lamp, however both species reproduced and developed egg masses containing eggs on pepper but not on cotton. Based on the reproductive factor pepper was a better host to *M*. *arenaria* isolates than to *M*. *floridensis*.

**Table 5.** Differential host test for characterizing *Meloidogyne floridensis* and *M. arenaria* isolates.

	Differential hosts^a^																	
	Tomato			Cotton			Peanut			Watermelon			Pepper			Tobacco		
*Meloidogyne* spp. and isolate code	GI^b^	EMI^b^	RF^c^	GI	EMI	RF	GI	EMI	RF	GI	EMI	RF	GI	EMI	RF	GI	EMI	RF
*M. floridensis*																		
Mf1	5.0	5.0	na	1.0	0	0	0	0	na	5.0	5.0	na	0	0	0.5	5.0	5.0	na
Mf2	5.0	5.0	na	3.8	0	0	0	0	na	5.0	5.0	na	0	2.6	0.8	5.0	5.0	na
Mf6	5.0	5.0	na	2.8	0	0	0	0	na	5.0	5.0	na	0	2.4	0.7	5.0	5.0	na
MfGnv14	5.0	5.0	na	0	0	0	0	0	na	5.0	5.0	na	0	2.0	0.9	5.0	5.0	na
*M. arenaria*																		
Ma1	5.0	5.0	na	0	0	0	0	0	na	5.0	5.0	na	0	5.0	6.9	5.0	5.0	na
Ma2	5.0	5.0	na	0	0	0	0	0	na	5.0	5.0	na	1.3	5.0	4.3	5.0	5.0	na
Ma3	5.0	5.0	na	0	0	0	0	0	na	5.0	5.0	na	0.2	5.0	9.0	5.0	5.0	na

Notes: ^a^Host plant cultivars used: *Solanum lycopersicum* cv. Agriset 334 (tomato), *Gossypium hirsutum* cv. Deltapine 16 (cotton), *Arachis hypogaea* cv. FL 07 (peanut), *Citrullus lanatus* cv. Charleston Grey (watermelon), *Capsicum annuum* cv. California Wonder (pepper), and *Nicotiana tabacum* cv. NC95 (tobacco). Data represent mean average of duplicate tests, each with three to five replicates. ^b^Galling (GI) and egg mass (EMI) indices were based on a 0 to 5 scale, where 0 = no galls or egg masses, 1 = 1-2, 2 = 3-10, 3 = 11-30, 4 = 31-100, 5 = ≥100 galls or egg masses per plant (Taylor and Sasser, 1978). ^c^Reproduction factor (RF) = ratio of nematode eggs at 60 days post inoculation to 3,000 second-stage juveniles initially used as inoculum (Sasser et al., 1984). na=not applicable.

### RKN infection of peach in commercial orchards

*Meloidogyne arenaria* (EST = A3, MDH = N3; Ma phenotype II) was found infecting Flordaguard in orchard A, and *M. floridensis* (EST = MF3, MDH = N1 phenotype) was found infecting Flordaguard in orchard B. These finding are consistent with identifications of RKN found in Florida (Brito et al., 2008, 2016). Second-stage juveniles and eggs from egg masses of both species were consistently extracted from soil and roots during a 7-month period at both sites (data not shown). The average number of J2 extracted per 200 cm^3^ soil at orchard A and B was 32 and 11, respectively, and eggs per gram of roots averaged 682 and 522 at orchards A and B, respectively. Other plant-parasitic nematodes species were found in soil collected from the peach rhizosphere. The following species were identified: Orchard A – *Mesocriconema ornatum* and *Pratylenchus hippeastri*, Orchard B – *M. xenoplax* and *P. brachyurus* (Inserra, pers. comm.). Although these nematodes were found in the soil, it was not established that they parasitized peach roots.

## Discussion

Sixty days from inoculation of *M*. *floridensis* MfGnv14 isolate on peach rootstock showed higher levels of infection on Flordaguard and Flordaglo than on Okinawa. Galling and egg mass indices were higher with inoculum levels of 5,000 and 10,000 compared with 2,000 eggs. Differences in susceptibility were more pronounced at increasing nematode concentrations. At the level of 10,000 eggs/plant, higher EGFR and RF were recorded on Flordaguard and Flordaglo, compared with Okinawa. A different trend was observed at the level of 5,000, wherein Flordaglo supported higher nematode reproduction than Flordaguard, and Okinawa. Differences were not observed at a level of 2,000. Previous reports have suggested that nematode infection increases with increases in inoculum densities (Di Vito et al., 2005; López-Pérez et al., 2006). In an earlier study GI was reported to be well-correlated with RF and the resistance threshold was set to GI = 2 (Maquilan et al., 2018a); thus, because the mean GI value is greater than 2 for all three peach cultivars, they would be classified as susceptible to MfGnv14, although Flordaguard and Flordaglo appeared to be better hosts than Okinawa. These results confirm earlier reports about the lack of resistance to some populations of *M. floridensis* in Flordaguard (Maquilan et al., 2018a), Okinawa (Sharpe et al., 1969), and Flordaglo (Chaparro, per. comm.). It is noteworthy that at a lower inoculum level (2,000 eggs), Okinawa had a GI = 1.5 and would have been (incorrectly) classified as resistant. It has been demonstrated that, for ligneous *Prunus* spp., a high and durable inoculum pressure (5,000 to 17,000 J2) would be necessary to obtain clear separation between hosts and nonhosts based on gall indices (Esmenjaud et al., 1992). Our findings suggest that a minimum of 5,000 eggs as inoculum would provide an acceptable level of accuracy after 2 months if galling or egg mass indices are used as a selection criterion.

Among the three peach rootstocks that were tested separately, only Lovell was susceptible to all *M. floridensis* isolates Mf1, Mf2, Mf4. Lovell supported high levels of nematode reproduction, consistent with previous findings (Stanley et al., 2009). The high rates of infection on Lovell and tomato showed that the inoculum was viable. The lack of infection on cv. Flordaguard was unexpected and may indicate inherent variability among populations of *M. floridensis*. Comparative studies of *M*. *floridensis* populations from different geographical locations and host plants is worthy of further investigations. The Mf4 isolate is the *M. floridensis* topotype originally collected from Nemaguard peach roots. Previously, this isolate was able to induce only 10 to 20% root galling on Flordaguard (compared to 80 to 100% in Guardian and Nemaguard peach rootstocks) after 1 year in microplots, which became the basis for considering Flordaguard as resistant to *M. floridensis* (Nyczepir et al., 2006). Several other *Prunus* species including some *P. cerasifera* accessions such as P.2980, P.2175, and P.1079 showed complete resistance (GI = 0) to the *M. floridensis* topotype (Esmenjaud et al., 1997, 2009; Lecouls et al., 1997; Rubio-Cabetas et al., 1998). In our experiment, the MfGnv14 isolate induced heavy infection on Flordaguard, which confirms previous results indicating that MfGnv14 isolate is a resistance-breaking variant of *M. floridensis* (Maquilan et al., 2018a).

Both Ma isolates induced galls and reproduced on Flordaguard, although Ma1 produced fewer galls and egg masses than Ma2. Ma1 caused galls on Okinawa, but no egg masses or eggs were recovered. Lovell supported a higher level of nematode galling and reproduction compared to Flordaguard. Okinawa appeared to be a poor host for *M. arenaria*, which might be due to a slower rate of development of juveniles in the roots or the inability of the nematode to complete its life cycle in Okinawa—a similar phenomenon was observed on Guardian when infected with *M. incognita* juveniles (Nyczepir et al., 1999). However, if galling intensity is used as a sole criterion for classifying resistance, a galling index that is greater than the resistance threshold (GI > 2) may not be practically significant for breeding when a source of high resistance (GI = 0) is needed in the development of new rootstocks (Maquilan et al., 2018a).

Peach rootstock Flordaguard was susceptible to the *M. floridensis* isolate MfGnv14 and both *M. arenaria* isolates, providing confirmatory evidence that these isolates overcome the RKN resistance in Flordaguard. Given that *M. floridensis* is a facultative meiotic parthenogenetic species capable of sexual reproduction under conditions of stress and crowding, genetic variation arising from cross-fertilization would facilitate an adaptive response to host-plant resistance (Handoo et al., 2004; Castagnone-Serena, 2006). When nematode resistance genes in RKN resistant rootstocks become nonfunctional it is imperative to seek new sources of resistance and understand the underlying mechanism of resistance against these nematode species to enhance breeding strategies.

Based on host differential tests, *M*. *floridensis* isolates Mf1, Mf2, Mf6 produced less marked root-galling on cotton, but failed to reproduce, whereas isolate MfGnv14, and *M*. *arenaria* isolates Ma1 and Ma3 did not produce galls nor reproduce on cotton. Based on the EMI as a reproductive parameter, tomato, watermelon, and tobacco were susceptible to *M. floridensis* and *M. arenaria*, whereas cotton and peanut were nonhosts. These findings with *M. floridensis* are consistent with those of a previous study (Stanley et al., 2009) of several *M. floridensis* isolates including Mf2 and Mf4 used in the present study but are not in agreement with the original description for *M. floridensis*, wherein tobacco was considered a nonhost (Handoo et al., 2004). *M. floridensis* produced pronounced root-galling on tobacco at inoculum levels of 3,000 freshly hatched J2 compared to an inoculum level of 5,000 eggs (Stanley et al., 2009) or 2,000 eggs (Handoo et al., 2004). Variability in host suitability outcomes after 60 days may be affected by differences in inoculum type, concentrations, and greenhouse conditions. It appears to be important to consider nematode developmental stage and concentration when choosing inoculum for an efficient screening method. On pepper, *M. floridensis* and *M. arenaria* isolates exhibited variability in their reproductive capabilities. The four *M. floridensis* isolates produced a low reproductive factor, whereas the three *M. arenaria* isolates produced a higher reproductive factor. The *M. floridensis* and *M. arenaria* isolates produced no readily visible galls on pepper, however, isolates Ma2 and Ma3 caused a trace of small galls. Based on these results cotton would be considered a nonhost (0.1 < RF <1.0), whereas pepper would be considered a good host to *M. arenaria* (RF ≥ 1).

Previous reports have been inconsistent regarding host suitability of pepper to *M. floridensis* either being reported as a good host (Stanley et al., 2009) or as a nonhost (Kokalis-Burelle and Nyczepir, 2004). Consistent with the latter study’s finding, the lower values for EMI and RF from the four *M. floridensis* isolates in the present study suggest that pepper cv. California Wonder is a poor host. In a growth chamber experiment at 28°C, *M. floridensis* was found incapable of infecting and reproducing on California Wonder 30 days after inoculation with 240 J2 (Maquilan et al., 2020). Of the six differential hosts, variations in reproductive potential among *M. floridensis* and *M. arenaria* isolates were observed only on pepper; therefore, additional hosts including major plant cultivars would be necessary to demonstrate greater differences among these isolates.

Three host races have been reported for *M*. *arenaria* in Spain (Fargette, 1987; Robertson et al., 2006). The three *M. arenaria* Florida isolates from peach reproduced on pepper but not on peanut, corresponding to the host range defined for race 3. This is the first report of the occurrence of *M. arenaria* race 3 in the United States. In addition to finding *M. arenaria* race 3 in Spain, the race has also been reported in Uruguay (Fargette, 1987; Robertson et al., 2006, 2009; Devran and Söğüt, 2011).

In two commercial peach orchards designated in this study, orchard A was infested with *M*. *arenaria*, and orchard B was infested with *M*. *floridensis*. RKN galls and egg masses were consistently recovered from peach roots sampled during the 7-month period, establishing that Flordaguard served as a host of this nematode species. There is little information regarding *M. arenaria* infecting and reproducing on peach in Florida, however, this pathogen is frequently encountered infecting other crops throughout the state.

In summary, the peach cv. Flordaguard was infected by *M. floridensis* (MfGnv14) and *M. arenaria* (Ma1 and Ma2). This was confirmed by testing of MfGnv14 recovered from the UF stone fruit breeding orchard and the two *M*. *arenaria* isolates on true to variety Flordaguard seedlings in greenhouse tests. In addition, both nematode species were found infecting the rootstock in commercial field plantings. These virulent isolates would be useful for future genetic studies to identify new sources of RKN resistance as well as to investigate the allelic relationships of RKN resistance loci such as *Mf* (conferring resistance to *M. floridensis*) in *Prunus kansuesis* (Maquilan et al., 2018a, 2018b) and *RMia* (conferring resistance to *M. arenaria* as well as *M. incognita*), which may be present in Okinawa (Duval et al., 2014). These would be critical research directions for future developments of improved rootstocks. The peach-originated isolates of *M. floridensis* and *M. arenaria* behaved variably on cotton and pepper with less marked root-galling on cotton and pepper, respectively.
